# Supplementary material for: Acylation of agricultural protein biomass yields biodegradable superabsorbent plastics
Source: Commun Chem. 2021 Apr 13;4:52. doi: 10.1038/s42004-021-00491-5 (PMC9814733; doi:10.1038/s42004-021-00491-5)
Supplement: Supplementary file 1 — Description of Additional Supplementary Files [file 42004_2021_491_MOESM1_ESM.pdf]

Description of Additional Supplementary Files

File Name: Supplementary Movie 1

Description: Fast water swelling of the PPC/25ED material
