# Supplementary material for: Acylation of agricultural protein biomass yields biodegradable superabsorbent plastics
Source: Commun Chem. 2021 Apr 13;4:52. doi: 10.1038/s42004-021-00491-5 (PMC9814733; doi:10.1038/s42004-021-00491-5)
Supplement: Supplementary file 2 — Supplementary Information [file 42004_2021_491_MOESM2_ESM.pdf]

Supplementary Information

Acylation of agricultural protein biomass yields biodegradable superabsorbent plastics

Antonio J. Capezza[1,2*], Faraz Muneer[2], Thomas Prade[3], William R. Newson[2], Oisik Das[4], Malin Lundman[5], Richard T. Olsson[1], Mikael S. Hedenqvist[1], and Eva Johansson[2*]

[1]KTH Royal Institute of Technology, Fibre and Polymer Technology Department, Teknikringen 56-58, SE-100 44, Stockholm, Sweden.

[2]SLU Alnarp, Plant Breeding Department, Box 190, SE-234 22, Lomma, Sweden.

[3]SLU Alnarp, Biosystems and Technology Department, Box 190, SE-234 22, Lomma, Sweden.

[4]Luleå University of Technology, Department of Civil, Environmental and Natural Resources Engineering, Structural and Fire Engineering Division, SE-97187 Luleå, Sweden.

[5]Essity Hygiene and Health AB, SE-405 03, Gothenburg, Sweden.

*Email: ajcv@kth.se, eva.johansson@slu.se

Number of figures: 6

Number of pages: 5

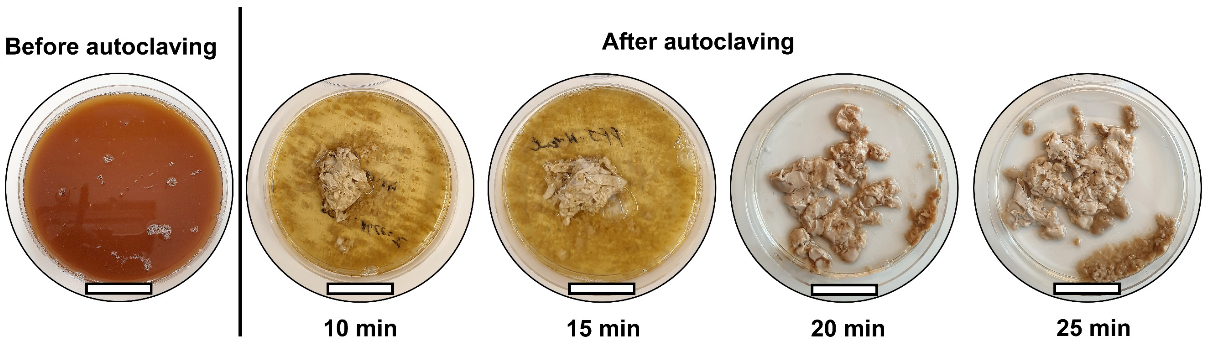

Supplementary Figure 1. Morphology of the PFJ before and after the autoclaving process. The samples are shown for the different autoclaving times performed on the PFJ. The scale bar in the photographs represents 2 cm.

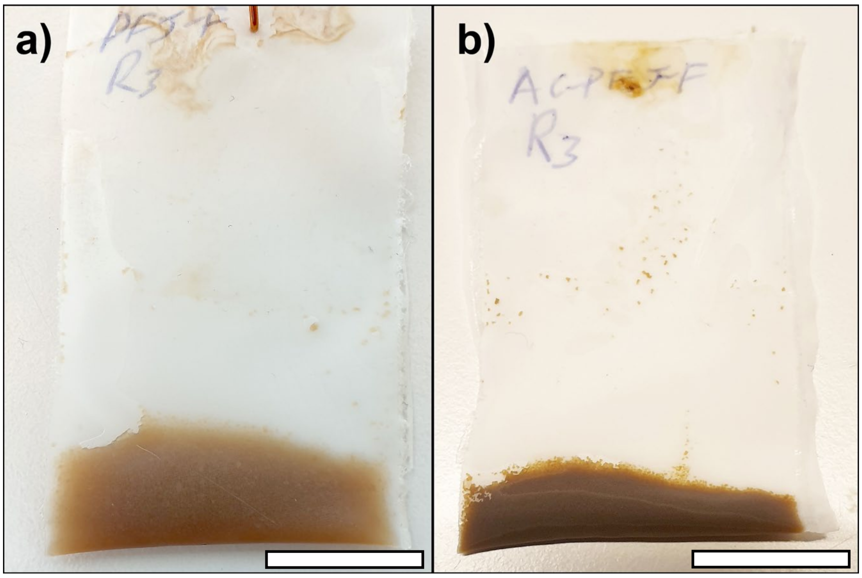

Supplementary Figure 2. Products after 60 min of water swelling, PFJ/25ED/A25 (a) and PFJ/A25/25ED (b). The scale bar in the photographs represents 2 cm.

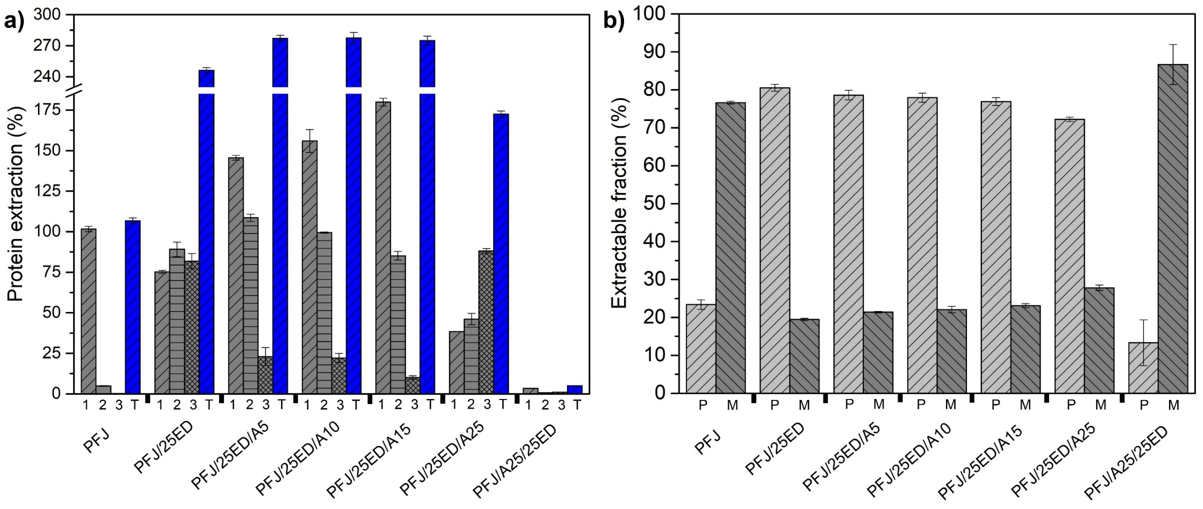

Supplementary Figure 3. Three-step (1-3) and total (T) protein extraction profiles (a) and the relation between polymeric (P) and monomeric (M) protein fractions (b) for the EDTAD functionalised potato fruit juice (PFJ) at different autoclaving times (A*X*). The total extractable proteins for the as-received PPC was used as the normalisation value.

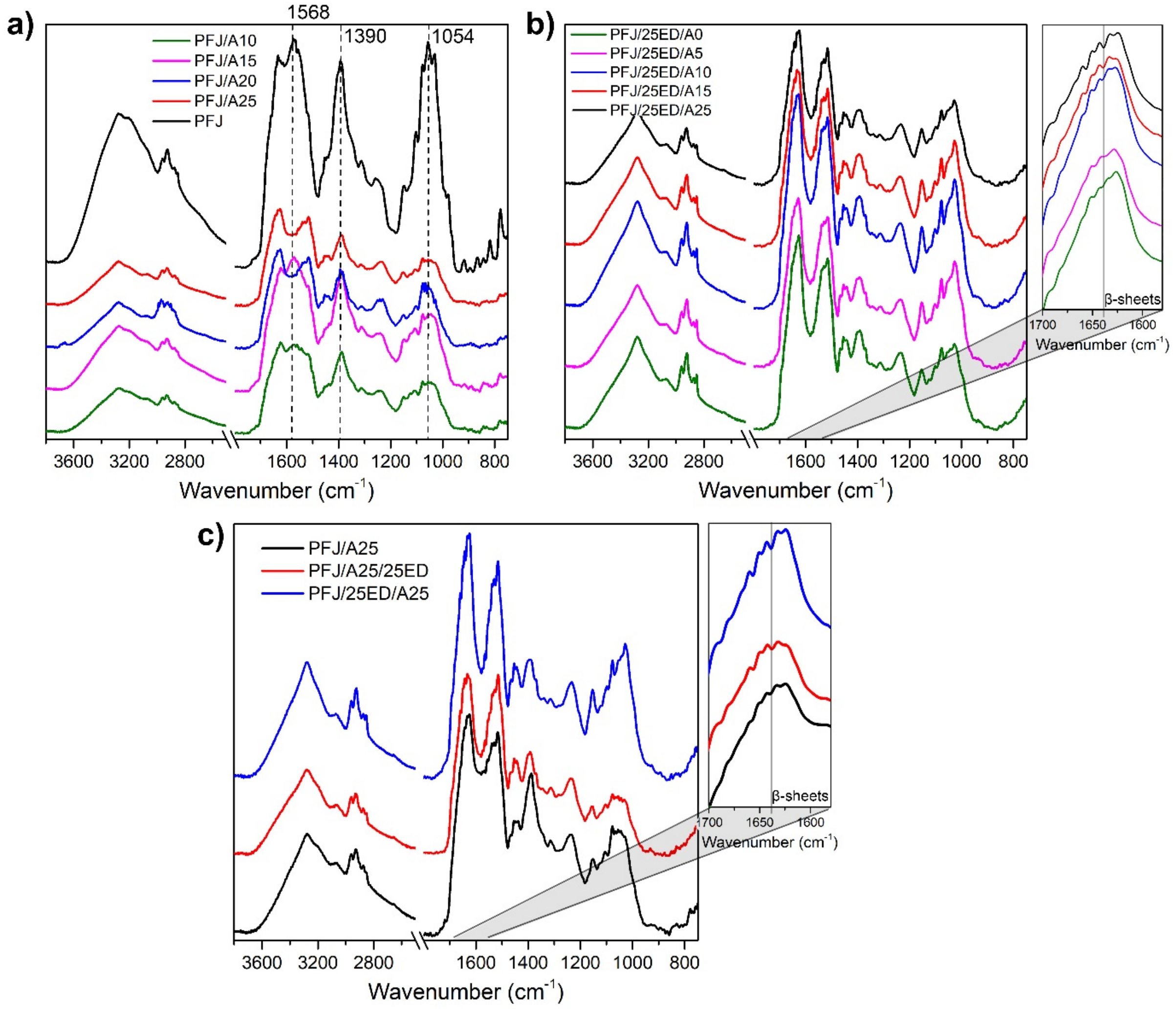

Supplementary Figure 4: FT-IR of the lyophilised autoclaved PFJ samples (a), EDTAD-functionalised PFJ and 25 min autoclaved samples (b), and a comparison between the solely 25 min autoclaved PFJ (PFJ/A25), autoclaved and EDTAD treated (PFJ/A25/25ED), and EDTAD treated and 25 min autoclaved (PFJ/25ED/A25) (c).

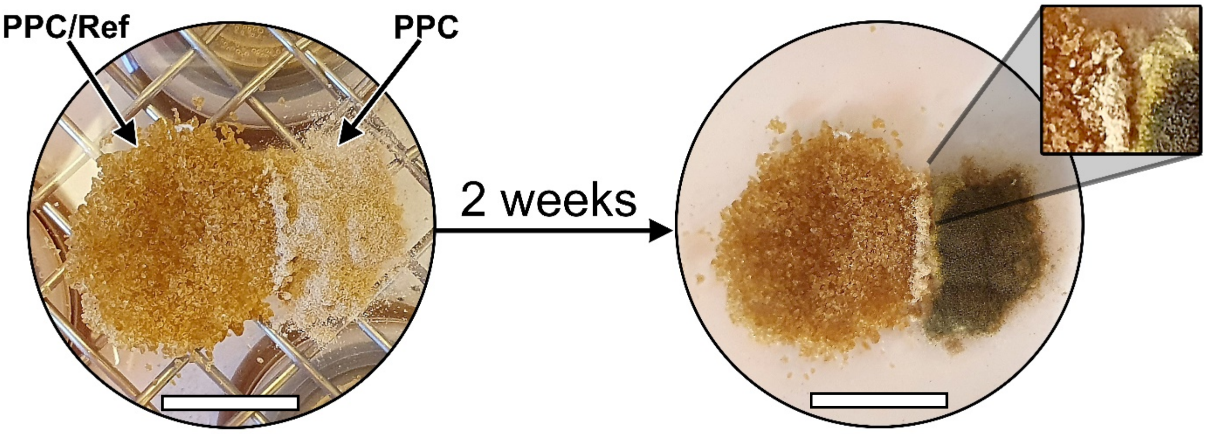

Supplementary Figure 5. Mould growing test of PPC/Ref in contact with as-received PPC powder. The samples were sealed in a container at 100 % RH for two weeks. No mould growth was visually apparent in PPC/Ref or around the sample's vicinity compared to PPC. The scale bar represents 2 cm.

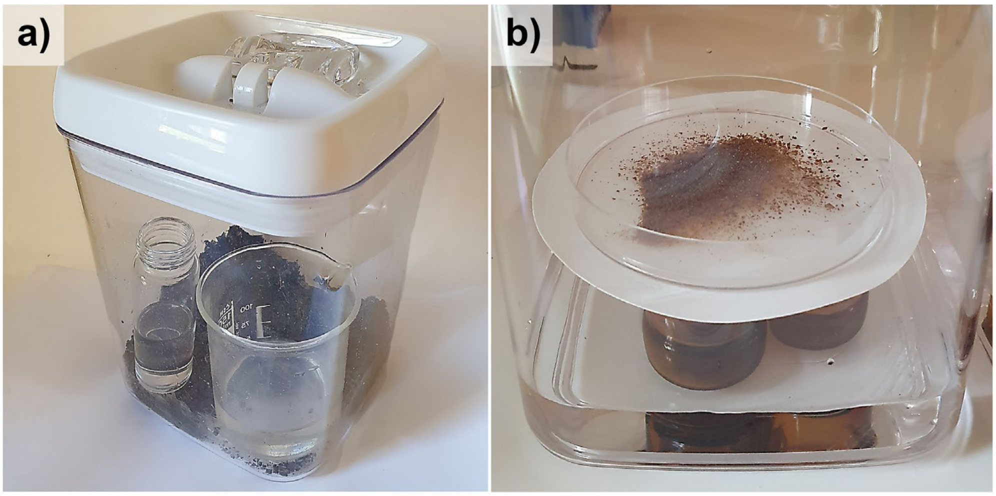

Supplementary Figure 6. Biodegradability-test box (a) and mould growth set-up (b).
